# Gemcitabine elaidate and ONC201 combination therapy inhibits pancreatic cancer in a KRAS mutated syngeneic mouse model

**DOI:** 10.21203/rs.3.rs-3108907/v1

**Published:** 2023-07-11

**Authors:** Ram Mahato, Virender Kumar, Bhartu Sethi, Dalton Staller

**Affiliations:** University of Nebraska Medical Center; University of Nebraska Medical Center; University of Nebraska Medical Center; University of Nebraska Medical Center

**Keywords:** Pancreatic cancer, Kras, ONC201, gemcitabine, PI3K/AKT, MEK

## Abstract

Approximately 90% of pancreatic cancer (PC) contain KRAS mutations. Mutated KRAS activates the downstream oncogenic PI3K/AKT and MEK signaling pathways and induces drug resistance. However, targeting both pathways with different drugs can also lead to access of toxicity. ONC201 targets DR5 to induce apoptosis in several types of cancers and has an excellent safety profile. ONC201 is also a dual PI3K/AKT and MEK pathways inhibitor. Gemcitabine (GEM) is a first-line chemotherapy in PC, but it is metabolically unstable, which can be stabilized by prodrug approach. Here, we used lipid-gemcitabine (L_GEM) conjugate, which is more stable and enters the cells by passive diffusion. We evaluated the efficacy of L_GEM and ONC201 in PanCan cells, and “KrasLSL-G12D; p53LoxP; Pdx1-CreER (KPC) triple mutant xenograft tumor-bearing mice. ONC201, in combination with L_GEM, showed a superior inhibitory effect on the growth of MIA PaCa-2 cells. ONC201 and L_GEM combination prevented neoplastic proliferation via AKT/ERK blockade, to overcome chemoresistance, and increased T-cell tumor surveillance. Simultaneous inhibition of the PI3K/AKT and MEK pathways with ONC201 is an attractive approach to potentiate GEM. Our findings provide insight into rational-directed precision chemo and immunotherapy therapy in PDAC.

## Introduction

Pancreatic ductal adenocarcinoma (PDAC) is one of the most difficult-to-treat cancers. The anti-metabolite GEM is the gold standard for the first-line treatment for PDAC (1). GEM works by various mechanisms; first, it inhibits the thymidylate synthetase, which is essential for inhibiting DNA synthesis and cell death (2). GEM is a prodrug that stepwise gets converted to two active metabolites, GEM diphosphate, and GEM triphosphate, by deoxycytidine kinase (3). GEM triphosphate, (difluorodeoxycytidine triphosphate) competes with endogenous dNTPs for incorporation into DNA. However, the GEM response rate is low, and overall survival has only improved minimally in PDAC (4). The main reason for this failure is its intrinsic instability due to rapid metabolism in the liver and early acquired resistance (5). Further, the intracellular uptake of GEM is affected by variable expression of human equilibrate nucleoside transporter-1 (hENT-1) receptors, in PC (6). Therefore, several efforts have been made to improve the overall performance of GEM in recent years. Various modifications have already been performed on the 4-(N)- and 5’-positions of GEM, such as the incorporation of poly(ethylene glycol) (PEG), valproic acid, 1,1’, 2-tris-nor-squalene acid (squalene) or valeroyl, heptanoyl, lauroyl, stearoyl linear acyl derivates in the 4-(*N*) site, and the addition of fatty acid chains or phosphate-function-protecting groups to the 5’-position (7) to improve its chemical stability. importantly, gemcitabine-5’-elaidate (CP-4126, C0–101) is a GEM prodrug consisting of a fatty acid derivative (abbreviated as L_GEM). L_GEM exerts its anticancer activity intracellularly and enters tumor cells independent of hENT1 (8). it was shown that L_GEM is not a substrate of metabolizing enzyme dCDA and retains its activity *in vivo* (9). However, the clinical study did not show an increased efficacy of L_GEM PC patients, indicating intrinsic resistance. Simultaneous activation of PI3K and ERK pathways have been suggested to generate resistance to GEM in PC patients (10, 11, 12). Another strategy to enhance GEM efficacy is combination therapy with other drugs. In a study from our group, GEM was combined with vismodegib, and miR-519c or miR-205, to assess their joined effect on cancerous cells (6, 13).

ONC201 is a new member of the imprisoned class of anticancer small molecules that primarily target dopamine receptor 2 (DR2) and TNF-related apoptosis-inducing ligand (TRAIL)-inducing activity (14). ONC201 also inhibits the death receptor 5 (DR5) pathway, which is upregulated in PC and correlates with poorer prognosis. DR5 targeting monoclonal antibody AMG 655 (conatumumab) has previously been investigated in a phase 2 clinical trial for treating PC in combination with GEM (15). Further, it has been suggested that cancer stem cells (CSCs) are responsible for tumor relapse and are highly resistant to chemotherapy and radiation (16). ONC201 has also demonstrated in eliminating CSCs in colorectal, glioblastoma, and prostate cancer models. ONC201 also inactivates AKT/ERK signaling in tumor cells and induces apoptosis (17).

Several preclinical studies have used a PC cell line-derived subcutaneous, orthotopic transplantation tumor, patient-derived subcutaneous/orthotopic xenografts (PDXs), and genetically engineered mouse models (GEMMs). However, to some extent, the cell line or PDX-derived immunodeficient mouse models do not reflect the actual growth environment of PC. It is believed that the GEMM model LSL-KrasG12D/+; Trp53fl/+, Pdx1-Cre (KPC) closely resembles pancreatic tumors (18). Further, the KPC model has demonstrated clinical relevance in predicting the activity of immunotherapy (19).

In this study, we combined L_GEM with ONC201 and evaluated it *in vitro* and in the syngeneic Kras mutated xenograft PC mouse model. During *in vitro* studies, the combination of ONC201 with L_GEM increased the G2/M phase in MIA PaCa-2 cells. We used KPC mouse-derived subcutaneous PC mouse models to evaluate the efficacy of these drugs. In the allograft study, the combination of L_GEM with ONC201 showed the best results, significantly reducing tumor growth, and a favorable toxicity profile was observed. In conclusion, the combinations of L_GEM with ONC201 showed a synergistic effect in inhibiting PC.

## Material and Methods

The details of materials and methods used are provided in the supplementary methods section.

## Results

### AKT and ERK pathways are active in PDAC tissues of patient samples.

**Figure 1:**
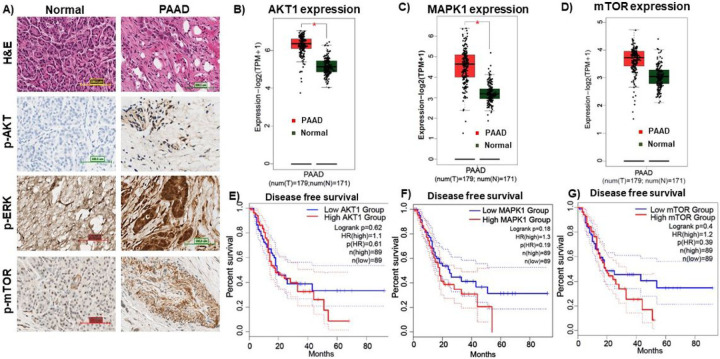
The AKT and ERK pathways are active in pancreatic adenocarcinoma (PAAD) tissue. A) Representative images of H&E, p-AKT, p-ERK, p-mTOR in PAAD cancerous tissue or its surrounding non-tumor tissue by immunohistochemistry. First panel show the tissue architecture of PAAD, second panel shows the representative images for p-AKT, third panel shows the p-ERK, and fourth panel shows p-mTOR expression in normal pancreatic tissues and PDAC (Magnifications: 40X). BC&D) On the GEPIA2 website, the expression levels of AKT1, MAPK1, and mTOR were compared between PAAD and a normal population (n=tumor 197; normal=171). AKT1 and mTOR genes had significantly higher expression levels in PAAD cancer specimens than in normal specimens (*P <0.01), whereas mTOR expression was not significantly different. Red color means tumor tissues and green color means normal tissues.

PDAC patient tissues showed a significantly higher levels of p-AKT, p-ERK, and p-mTOR in PDAC cancerous tissue compared to their surrounding non-tumor tissues (Fig. 1A). The first panel shows the tissue architecture of PDAC, the second panel shows a representative IHC images of p-AKT, the third panel shows p-ERK, and the fourth panel shows a representative image of p-mTOR expression in normal pancreatic tissues and PDAC.

Using the GEPIA2 website, AKT1, MAPK1, and mTOR expression levels were compared between PDAC and a normal population (for tumor tissues, n = 197, and for normal tissues, n = 171) (Fig. 1B–D). AKT1 and mTOR genes had significantly higher expression levels in PDAC cancer samples than in normal samples (*P< 0.01), whereas mTOR expression was not significantly different. In Fig. 1, red color denotes expression in tumor tissues and green color denotes expression in normal tissues.

### L-GEM and ONC201 synergistically inhibited pancreatic cancer cell proliferation in vitro

**Figure 2:**
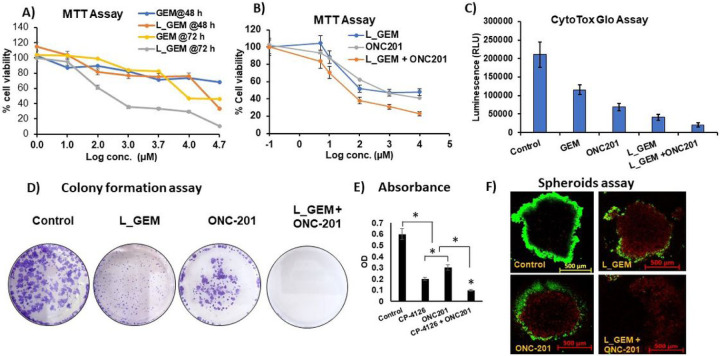
Combination of ONC201 with GEM analog (L_GEM) efficiently reduces the viability and tumorigenic potential of pancreatic cancer cells MIA PaCa-2. A) MTT-based cytotoxicity assay of gemcitabine (GEM) and gemcitabine elaidate (CP-4126, L_GEM) at different concentrations and time points. MiA PaCa-2 cells were treated with increasing concentrations of compounds for 48 h, or 72 h. Percentage cell viability was measured by using MTT, in which DMSO treated cells served as a control (100%). B) Cells were exposed to ONC201 in combination with L_GEM for 72 h then cell viability was determined by MTT assay. (Data are expressed as mean ± SD (n=5) * P<0.05). C) CytoTox glo assay after 48 h treatment with drugs. D) Colony formation assay: MIA PaCa2 cells treated with treatments formed less colonies than control cells. (E) The columns represent the mean colony number for each group from at least three independent experiments. Data are expressed as mean ± SD. F) Tumor spheroid assay: cells were exposed to ONC201 in combination with L_GEM for 48 h then allowed to grow for 14 days. Calcein AM assay was used to distinguish live (green) and dead cells (red).

We compared the cytotoxicity effects of lipid-GEM conjugate (L_GEM) with GEM in MIA PaCa-2 cells utilizing an MTT assay. Each of the drugs displayed a dose-dependent cytotoxic effect (Fig. 2A). After 48 h of GEM exposure to MIA PaCa-2 cells, the half inhibitory concentration (IC_50_) was observed at 10 ± 1 μM (Fig. 2A). At this point, MIA PaCa-2 cells exposed to L_GEM showed an IC_50_ concentration of 1.0 ± 0.2 μM. After 72 h of treatment, a 50% reduction in cell viability compared to control cells was achieved at 1 μM and 340 nM for GEM and L_GEM, respectively. At both of these time points, the cytotoxicity of L_GEM was significantly higher than GEM.

We performed a cytotoxicity assay on MIA PaCa-2 cells using L_GEM and ONC201 combination therapy. We observed that L_GEM was more toxic than ONC201 at all tested concentrations (**Fig. S1A**). Further, we observed significantly enhanced cytotoxicity in combination treatment compared to the single drug at 72 h (Fig. 2B, IC_50_ = 200 nM *p < 0.05). Further, combination index values between L_GEM and ONC201 were calculated using Chao and Talalay method. CI values are < 1 for combination at all concentrations, indicating the *in vitro* synergism between L_GEM and ONC201 in PC cells (**Fig. S1B**).

In the CytoTox Glo^™^ Assay we observed that both L_GEM and ONC201 treatment induced high cytotoxicity at their IC_50_ concentrations (Fig. 2C). Intriguingly, treatment with combination led to much higher levels of death-cell protease release in MIA PaCa-2 cells. The combination of L_GEM and ONC201 inhibited colony formation, tumor spheroid growth, and invasion of MIA PaCa-2 cells more effectively than either drug alone. Figure 2D shows that there were almost no colonies in the samples treated with the combination of L_GEM and ONC201, while ONC201 alone also showed a significant decrease in the number of colonies compared to non-treated samples. Figure 2E shows that compared to the control group (OD 0.6 ± 0.01), the OD values of monotherapy treatment groups were 0.2 ± 0.02; combination treatment inhibited the colony formation of cells better than either drug alone, which was consistent with the results from the cell viability assays.

Tumor spheroids are formed by CSCs within a tumor/cancer cell line and are correlated with cancer metastasis and aggressiveness (20). An evident disruption of the architectural structure of the spheroid population was observed in the tumor spheroids exposed to L_GEM and ONC201 compared to control spheroids. All the treated spheroids also showed a decrease in cell viability, as indicated by decreased Calcein AM staining (green) and increased propidium iodide (PI) staining (red) (Fig. 2F). However, the combination exhibited the highest cytotoxicity resulting in dead cells distributed across the inner core of spheroids and even disruption of spheroid structural integrity.

### L_GEM and ONC201 combination arrests MIA PaCa-2 cells in the G2 phase and induces apoptosis more effectively than their individual drugs.

**Figure 3:**
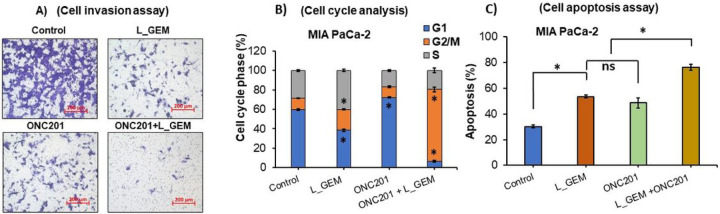
Treatment with ONC201 and L_GEM efficiently reduces invasion and induces apoptosis of MIA PaCa-2 cells. A) Matrigel invasion assay: After treatment with L_GEM and its combination with ONC201, significantly reduced the population of the invaded cells in MIA PaCa-2 cells. B) Data from cell cycle assay by flow cytometry using MIA PaCa-2 cells. There was a significant difference in the cell cycle arrests in L_GEM in combination with ONC201 compared with untreated cells. Data are expressed as mean ± SD. (C) Data for apoptosis assay suggest that combination treatment significantly induced apoptosis in MIA PaCa-2 cells.

L_GEM, ONC201, and their combination decreased the extent of migration in MIA PaCa-2 cells across the Transwells insert membrane compared to control cells. (Fig. 3A). However, the combination treatment significantly decreased migration compared to other groups.

Cell cycle analysis suggests that upon treatment of MIA PaCa-2 cells with combination of L_GEM and ONC201, they were found mostly in the G2 phase (Fig. 3B, S2). The G2 phase arrest occurs due to various factors such as DNA damage, insufficient nutrients, or signaling from growth factors. The cells will remain in the G2 phase until the issue is resolved and normal cell division can resume. If the damage is irreparable, the cells may undergo apoptosis. In the untreated control cells, the percentage of cells in the G1, G2/M, and S phases were 59.72 ± 8.6%, 11.82 ± 3%, and 28.42 ± 5.6%, respectively. We found that upon L_GEM treatment, 40.3 ± 4.6% of cells were arrested in the S Phase, while 21.37 ± 6.2% were found in and G2 phase (Fig. 3B). After treatment with ONC201, 72.31 ± 7.6% of cells were found in the G1 phase, whereas their population in G2/M and S phases was 10.93 ± 2.2% and 16.71 ± 3.2%, respectively. In combination with treated cells, we surprisingly observed 74.12 ± 11.6% of the population in the G2 phase, while 6.53 ± 1.5% and 19.36 ± 5.6% cells were observed in the G1 and S phases, respectively.

Apoptosis is a crucial regulator of tumor growth (9). L_GEM and ONC201 were found to induce apoptosis of cancer cells in a dose-dependent manner; therefore, we next evaluated the effects of L_GEM combined with ONC201 on apoptosis of MIA PaCa-2 cells (**Fig. S3**). As shown in Fig. 3C, after 24 h of drug treatment, the combination induced apoptosis more significantly than single agents. In the untreated control MIA PaCa-2 cells, the percentage of Annexin V-positive cells was 30.13 ± 2. In cells treated with L_GEM and ONC201 alone, these percentages increased to 53.5 ± 5.35 and 48.6 ± 8.3, respectively. In cells treated with the combination of L_GEM and ONC201, the apoptotic percentage was 76.30 ± 8.6.

**Figure 4:**
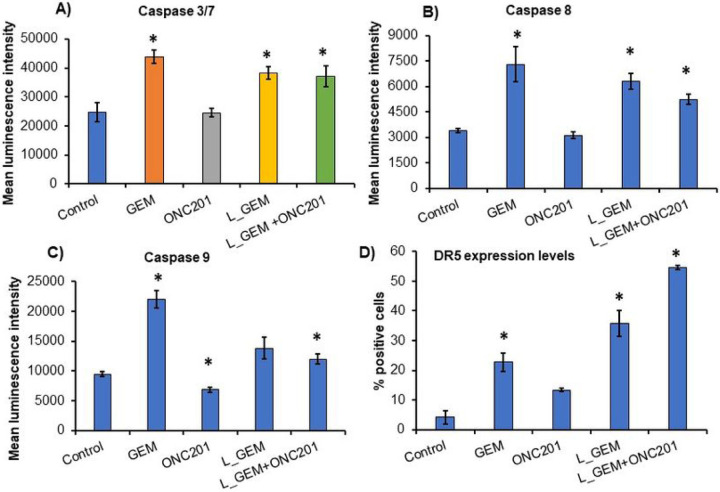
L_GEM and ONC201 induce cell apoptosis by different mechanisms. **A-C)** Caspases activity assay. MIA PaCa-2 cells were treated with various compounds for 24 h and mean luminescence intensity reflecting Caspase 3/7, Caspase 8 and Caspase 9 activities were measured. D) Cells after treatment with various compounds were analyzed for DR5 expression using Flowcytometry (n=3, *p<0.05).

In Caspase 3/7 activity assay we observed that GEM and L_GEM induced ΔMLI values of 43,826 ± 2259 and 38,298 ± 2055, respectively indicating higher Caspase 3/7 activity at their IC_50_(Fig. 4A–B), ONC201 did not affect this activity (ΔMLI 24,486 ± 1431). Therefore, in the group treated with L_GEM combination with ONC201 (at IC_50_ levels), the ΔMLI value 37,079 ± 3526 was not significantly different from L_GEM alone treatment (Fig. 4A). These results support the hypothesis that ONC201 treatment does not induce apoptosis but lowers the threshold for the apoptotic pathways in MIA PaCa-2 cells. Similarly, Caspase 8, and 9, which are upstream of Caspase 3 and 7, were also not induced by ONC201, while GEM and L_GEM significantly enhanced their activity compared to control cells (Fig. 4B&C). These results indicate that ONC201 reduces tumor cell growth independent of apoptosis.

GEM is known to enhance TRAIL-induced cell death by upregulating DR5. Due to ATF4/CHOP-mediated integrated stress response (ISR) pathway activation by ONC201, which results in DR5/TRAIL-mediated apoptosis. In vehicle-treated cells, DR5 positive cell population percentage was 4.1 ± 2.2. In GEM-treated cells, it was 22.8 ± 3.1, whereas in L_GEM-treated cells, it was 35.9 ± 4.3 (Fig. 4D). The DR5 positive cell population increased by 13.1 ± 0.5% with ONC201 treatment alone and by 54.6 ± 0.7% with L_GEM treatment, indicating a modest impact of ONC201 on DR5 expression.

#### Mapping of bioenergetics in pancreatic cancer cells

**Figure 5:**
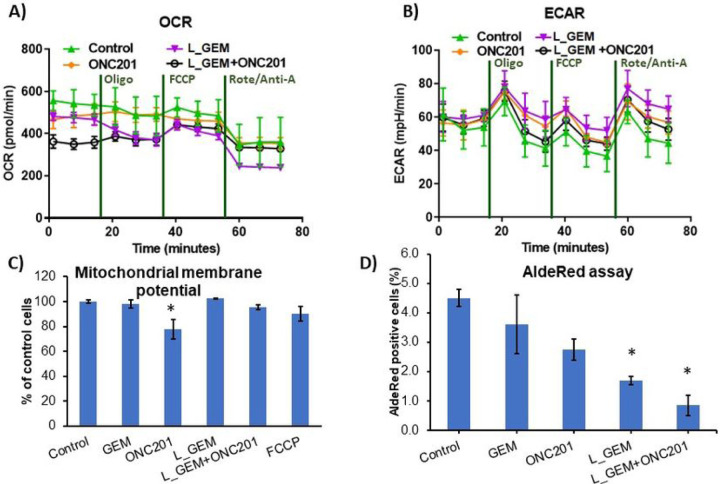
Mitochondrial stress test and glycolysis test in drug treated MIA PaCa-2 cells. To evaluate mitochondrial function, cells were injected with oligomycin (oligo), carbonyl cyanide-4-(trifluoromethoxy)phenylhydrazone (FCCP), and rotenone/antimycin-A. A) Quantification of the basal oxygen consumption rate (OCR), proton leak, maximal OCR, ATP-linked OCR, spare capacity in Ctr and treated mitochondria. B) Extracellular acidification rate with or without treatments for 24 h Values are expressed as mean ± SEM of three independent experiments conducted in triplicate on each treatment group. C) Mitochondrial potential determined by flow cytometry - Tetramethylrhodamine, ethyl ester, perchlorate (TMRE) staining. ALDH positive cell percentage in MIA PaCa-2 cells post 24 h treatment with various compounds (n=3, *p<0.05).

To better understand the change in metabolic and energetic requirements of MIA PaCa-2 cells after treatments, cells were analyzed in real-time using a Seahorse Extracellular Flux (XF) 96-well Analyzer. Both cellular oxygen consumption rate (OCR), resulting from oxidative phosphorylation (OXPHOS), and extracellular acidification rate (ECAR), associated with glycolytic metabolism (glycolysis), were simultaneously monitored (Fig. 5A&B). Results indicate that MiaPaCa-2 cells are highly glycolytic.

We determined whether the membrane potential of mitochondria is affected by treatments (21). We observed that the fluorescence intensity of mitochondria was not affected by GEM or its analog L_GEM (Fig. 5C). However, in cells treated with ONC201, the fluorescence intensity of mitochondria was reduced to ~ 80% compared with the controls. interestingly, we found that cells treated with the combination of L_GEM and ONC201 showed slightly less loss in mitochondrial membrane potential than cells treated with ONC201 alone. Many PC cell lines, including MIA PaCa-2, and BxPC3 cells, also contain an AldeRed + population that displays stem cell properties [23]. We therefore determined the effects of drug treatments on the proportion of AldeRed + cells in vitro (Figure S4). Treatment with ONC201 (2.75 ± 0.6%) and L_GEM (1.70 ± 0.3%), but not with GEM (3.6 ±1.1) significantly decreased (P < 0.01) the percentage of AldeRed + cells (Fig. 5D). The percentage of AldeRed^+^ cells in L_GEM and ONC201 combination treated group was further significantly dcreased (*p* < 0.01) to 0.85 ± 0.1%.

### L_GEM and ONC201 combination effectively inhibits the growth of KPC tumor-bearing syngeneic mice.

**Figure 6:**
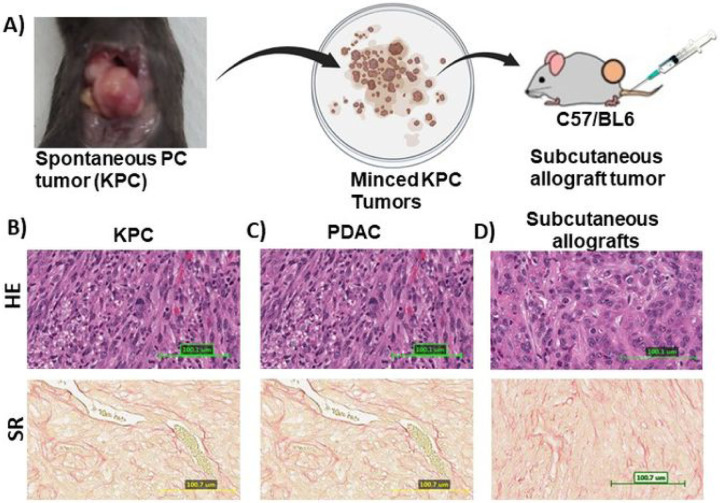
Schematic representation of in vivo drug testing using syngeneic subcutaneous KPC allografts. A) Tamoxifen induced spontaneous tumor bearing KPC mouse. The harvested tumor was grafted subcutaneously in C57/BL6 mice flank and randomized for treatments. Representative image of Hematoxylin and eosin, and Sirius red staining of KPC tumors (magnification, 20×; scale bar, 100 μm).

**Figure 7:**
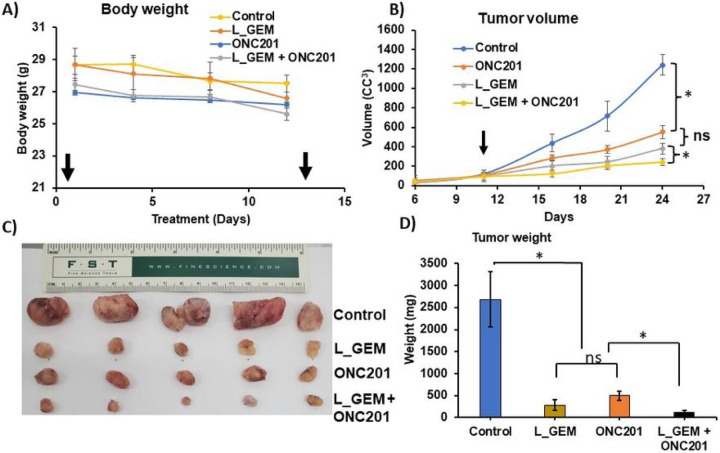
L_GEM and ONC201 combination therapy more effectively inhibits tumor growth compared to their monotherapies after systemic administration in KPC pancreatic cancer allograft bearing mice. (A) Effect of treatments on body weight change. The weight of each mouse was measured once a week. (B) Effect of therapy on tumor volume (n=5). The volume of each tumor was measured every week. The average tumor volume in the control or L_GEM (20 mg/kg), ONC201 (20 mg/kg), or their combination treated group was plotted (mean ±SD, n=5, * p <0.05). C) Tumors were harvested from different treatment groups after completion of the therapy. D) Tumor weight (mean ±SD, n=5, *p<0.05).

We used PDAC tumor tissues from KPC mice to create syngeneic subcutaneous tumors (Fig. 6A). KPC mouse tumors recapitulate many of the salient clinical and histopathological features of human disease (Fig. 6B–D). Animals bearing tumors were treated with doses of L_GEM and ONC201 at 20 mg/kg and 15 mg/kg, respectively. The selected doses showed no severe toxicity, as evident by the appearance, physical activity, and body weights of treated animals (Fig. 7A).

**Figure 8:**
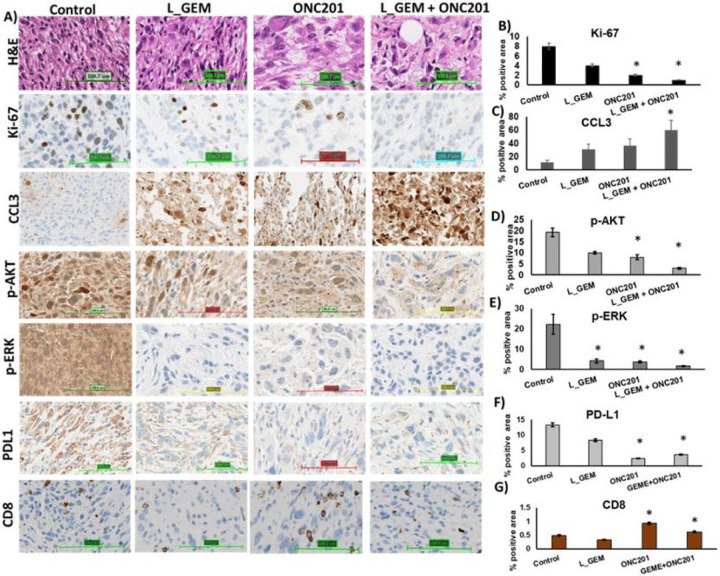
Immunohistochemistry (IHC) staining of mice tumor samples. After systemic delivery in KPC pancreatic cancer allograft carrying mice, treatment with the combination of L_GEM and ONC201 was found to reduce tumorigenic genes more effectively than each of the individual treatments. A) Representative HE, Ki-67, CCL3, p-AKT, p-ERK PDL1, and CD8 staining from tumor tissues from treatment groups. All images were obtained at 40×. Quantification of positive staining of B) Ki-67, C) CCL3, D) p-AKT, and E) p-ERK F) PDL1, and G) CD8 from the samples (n=3, *p< 0.05 compared to control).

As shown in Fig. 7B, a significant increase in the tumor volume occurred on the 11th day after transplantation. Consistent with cell viability, cell proliferation, and apoptosis experiments, the *in vivo* efficacy study showed that L_GEM and ONC201 monotherapies had no significant effect on tumor growth inhibition (Fig. 7B). Compared to L_GEM monotherapy, the combination of ONC201 and L_GEM significantly reduced the tumor size (Fig. 7C–D). Representative tumor images of isolated tumors are shown in Fig. 7C. The number of Ki67-positive cells in tumor tissues of both control and treatment groups showed that the combination significantly suppressed tumor cell proliferation (Fig. 8A&B). in addition, ONC201 inhibited the expression of p-ERK ^(T202/T204)^ and p-AKT ^(Ser473)^ in tumors and upregulated the expression of cleaved Caspase 3, thereby inhibiting the growth of these breast cancer cells *in vivo* (Fig. 8A&8C, D, E).

The PD-1 receptor (programmed death 1) is primarily expressed on T cells, and its physiologic interaction with PD-L1 on cancer cell results in T cell function suppression (22). Kras is in pancreatic cancer cells is known to promotes the expression of PD-L1 through reactive oxygen species (ROS)- mediated growth factor signaling (23). So, we were interested in assessing how ONC201 might affect PD-L1 expression and subsequent CD8 cell tumor invasion. We observed a significant decrease in PD-L1 expression following treatment with ONC201, but not with L_GEM (Fig. 8&).

We next tested whether L_GEM and ONC201 might have different impacts on T cell functions such as their ability to infiltrate the tumor tissues. As shown in Fig. 8A&8G, administration of ONC201 alone significantly increased the number of tumor infiltrating CD8 + T lymphocytes compared to the control samples. L_GEM treatment significantly reduced the number of CD8 + T cells in the tumor tissues, whereas their combination did not show such suppressive effect.

## Discussion

Only 15–20% of patients with pancreatic cancer are eligible for surgical treatment because most of these patients have advanced or metastatic diseases. Chemotherapy is therefore the only treatment available to most people with PC. However, the development of chemoresistance often leads to poor therapeutic outcomes. Several combination therapies have been tested to overcome GEM resistance, but most of them have shown mixed results at the best. Among these, only two combination treatments, one is 5-fluorouracil (5-FU)/leucovorin with irinotecan, oxaliplatin (FOLFIRINOX), and the other is GEM with nab-paclitaxel have proven to be the best for metastatic PC. However, the effectiveness of these chemotherapies is severely constrained by high-grade toxicity and the emergence of chemoresistance.

The underlying mechanisms for the development of GEM resistance revolve around the upregulation of transcription factors, enzymes, and signaling pathways, involved in nucleoside metabolism. Cumulative evidence from past studies suggests that enhancing GEM metabolic stability and reducing its non-specific distribution is a viable strategy for increasing effectiveness and reducing side effects. GEM’s rapid degradation could be prevented by bioconjugation and nanomedicine techniques. We have previously shown that GEM’s efficacy could be improved by decreasing its metabolism, controlling its accumulation at the tumor site, and rationally combining it with chemosensitizing drugs (13, 24, 25, 26). GEM loading into nanoparticles is difficult, and bioconjugation with large molecules like polymers is a complex process that may face issues with the scaling up. Another bottleneck for GEM’s efficacy is its uptake by tumor cells. GEM is a hydrophilic nucleoside, and its cellular uptake depends on membrane nucleoside transporter hENT1 expression. L_GEM (gemcitabine-5’-elaidic acid ester) prodrug used in this study is a GEM analog that, by virtue of a fatty acid conjugate, protect it from rapid degradation and can enter cells in a transporter-independent manner (27).

To explain the decreased cell survival observed during co-treatment with ONC201 and L_GEM, we reasoned that ONC201 might lower the apoptotic threshold. We investigated this by measuring the activities of Caspases 3, 7, 8, and 9 in MIA PaCa-2 cells after 48h of treatment. The intrinsic apoptotic pathway is initiated by Caspase 9 by inducing mitochondrial stress. On the other hand, Caspase 8 mediates the extrinsic apoptotic pathways that are mainly activated by cell surface death receptors. These two initiator Caspases (8 and 9) activate the executioner Caspases 3, and 7, which deteriorate cell structures to induce apoptosis (28). Compared to vehicle treatment, ONC201 treatment did not affect Caspase 3/7 activities in cells, but GEM or L_GEM treatment caused a significant increase of Caspase 3/7 activities (Fig. 4A). Further, GEM/L_GEM significantly enhanced Caspase 8 (external apoptosis) and 9 (internal apoptosis) activities in MIA PaCa-2 cells but ONC201 did not show any induction of Caspase activity (Fig. 4B&C). Similar observations were reported earlier when doxycycline did not affect the Caspase activity but primes cancer cells for apoptosis by GEM (29). It also implies that ONC201 executes cell death by some mechanisms other than Caspase activation, which is also reported by Greer et al., in breast cancer cells (30).

The TNF family member TNF-related apoptosis-inducing ligand (TRAIL) is a potent inducer of apoptosis. TRAIL binds to DR5 and activates Caspase-8-initiated apoptosis (31). GEM has been linked to a synergistic cytotoxic effect with TRAIL and it was found that pretreatment with GEM enhanced TRAIL-induced apoptosis accompanied by DR-5 up-regulation (32).

The overexpression of several IAP family proteins, including cIAP1 and XIAP in pancreatic cancer cells makes them resistant to TRAIL-induced extrinsic apoptosis. cIAP-1 and XIAP inhibit TRAIL-induced apoptosis by preventing the cleavage of Caspase 3, 7, or 9, thereby preventing subsequent apoptotic events. These cells can, however, still undergo type II extrinsic apoptosis, which amplifies cell death signaling via the mitochondria. Further, our results indicate that GEM or L_GEM treatment increased the percentage of DR5-positive cell population in MIA PaCa-2 cells (Fig. 4D). These findings also imply that the combination can be used successfully as chemotherapy to overcome TRAIL-resistant defects in MIA PaCa-2 cells by increasing DR-5 expression.

Further, several signaling pathways are involved in GEM resistance, including PI3K/AKT, MAPK signaling, and IL-6/IL-6R/STAT3 pathways (33). More than 90% of pancreatic cancers have mutant *K-Ras that* activates various downstream effector-signaling pathways, including the PI3K/AKT. Further, the PI3K pathway responds to stimuli from multiple growth factor receptors present on the cancer cell surface (34). A downstream element of this pathway is the mammalian target of rapamycin (mTOR). The PI3K/AKT/mTOR pathway is essential for many cellular functions, including growth, survival, and proliferation. Also, PI3K signaling in stromal cells modifies the tumor microenvironment to dictate disease outcomes. The high incidence of mutations in the PI3K signaling cascade, accompanied by activation of parallel signaling pathways, makes PI3K a promising candidate for drug therapy. Cui et al. demonstrated that treatment with the mTOR inhibitor everolimus inhibits the growth and activity of pancreatic cancer resistant to GEM (35). Similarly, Gu et al. showed that miR-3178 inhibitor reduced GEM resistance in PC cells by inhibiting PI3K/AKT pathway (36). In addition, Zheng et al. found that ERK1/2 activity also protects PC cells from apoptosis caused by chemotherapy. Using an ERK1/2 inhibitor (U0126) in combination with GEM, they showed synergistic therapeutic effects at lower doses of GEM (37).

The RAS/RAF/MEK/ERK and PI3K/AKT/mTOR signaling pathways interact and act as a compensatory mechanism when one pathway is inhibited. This supports the rationale for inhibiting both pathways simultaneously (38, 39). Awasthi et al. recently demonstrated that combining the PI3K inhibitor MK2206 and the MAPK inhibitor trametinib with chemotherapy enhanced the antiproliferative effect in a PDX-derived preclinical PDAC tumor model (40). Our study demonstrates that simultaneous inhibition of the PI3K and ERK pathways induce higher toxicity to L_GEM in MIA PaCa-2 cells.

Immunotherapies that induce T-cell responses have demonstrated efficacy against a subset of solid tumors in patients and rodents, but these treatments are ineffective against PC (41). There may be several causes, including desmoplasia, an immunosuppressive microenvironment (TME), and excessive PDL-1 expression, among others. Kras hyperactivity has recently been demonstrated to upregulate PDL-1 expression in cancer cells, rendering these malignancies resistant to T cell therapy (42). T cell migration, on the other hand, was demonstrated to be greatly reduced in vitro and in vivo by GEM, explaining the poor clinical result of combined PD-1 antibody therapy in PC. Our findings are consistent with prior studies, and ONC201, but not L_GEM therapy, decreased PDL1 expression due to inhibition of KRAS signaling. Furthermore, whereas L_GEM alone prevented T cell migration into tumors when combined with ONC201, T cell existence was restored (Fig. 8).

In summary, we have shown that simultaneous inhibition of ERK/PI3K improves the effectiveness of the current chemotherapy in PC. We combined L_GEM with ONC201 and evaluated it *in vitro* and the syngeneic Kras mutated xenograft PC mouse model. We used KPC mouse-derived subcutaneous models of pancreatic cancer to evaluate. In the allograft study, the combination of L_GEM with ONC201 demonstrated a favorable toxicity profile and significantly reduced the tumor growth, compared to their monotherapy. Furthermore, L_GEM with ONC201 had a favorable effect in inducing T-cell infiltration and inhibiting PC tumor development.

## Data Availability

The results shown in this manuscript were partially based upon data from Gene Expression Profiling Interactive Analysis 2. The experimental data that support the hypothesis within this paper are available from the corresponding author upon reasonable request.
